# Iron Speciation and Iron Binding Proteins in *Arthrospira* *platensis* Grown in Media Containing Different Iron Concentrations

**DOI:** 10.3390/ijms23116283

**Published:** 2022-06-03

**Authors:** Gloria Isani, Alberto Niccolai, Giulia Andreani, Thomas Dalmonte, Elisa Bellei, Martina Bertocchi, Mario R. Tredici, Liliana Rodolfi

**Affiliations:** 1 Department of Veterinary Medical Sciences, Alma Mater Studiorum-University of Bologna, 40064 Bologna, Italy; gloria.isani@unibo.it (G.I.); thomas.dalmonte2@unibo.it (T.D.); martina.bertocchi3@unibo.it (M.B.); 2 Department of Agriculture, Food, Environment and Forestry (DAGRI)-University of Florence, 50144 Florence, Italy; alberto.niccolai@unifi.it (A.N.); mario.tredici@unifi.it (M.R.T.); liliana.rodolfi@unifi.it (L.R.); 3 Proteomic Laboratory, Department of Surgery, Medicine, Dentistry and Morphological Sciences with Transplant Surgery, Oncology and Regenerative Medicine Relevance, University of Modena and Reggio Emilia, 41124 Modena, Italy; elisa.bellei@unimore.it

**Keywords:** *Arthrospira platensis*, cyanobacteria, culture media, iron bioaccumulation, iron speciation

## Abstract

Cyanobacteria are characterized by high iron content. This study investigated the effects of varying iron concentrations (1, 5, and 10 mg L^−1^) in the culture media on the biochemical composition and the iron bioaccumulation and speciation in *Arthrospira platensis* F&M-C256. Iron content measured in biomasses varied from 0.35 to 2.34 mg g^−1^ dry weight depending on the iron concentration in the culture media. These biomasses can be considered of interest for the production of spirulina-based supplements with low and high iron content. Iron speciation was studied using size exclusion chromatography followed by atomic absorption spectrometry and proteomic analysis. The role of C-phycocyanin as an iron binding protein was also investigated. Overall, the present results provide a better understanding of iron metabolism in cyanobacteria and a foundation for further studies.

## 1. Introduction

The cyanobacteria of the genus *Arthrospira*, commonly known as spirulina, are characterized by an excellent nutritional profile with high digestible protein content (60–70%) [1,2]. They are also considered an interesting source of macro (Ca, Mg, P) and trace (Fe, Zn, Cu, Se) elements, vitamins, γ-linolenic acid, sulfated polysaccharides, phycocyanin, and bioactive molecules [3]. The possibility of modifying the biochemical composition of algal biomass by varying the growing conditions is an attractive opportunity to obtain products with enhanced nutraceutical properties. In particular, the presence of high concentrations of iron could be an interesting opportunity for people subjected to an inadequate dietary iron intake.

Iron performs important functions in biological systems; however, its redox activity, essential for many biochemical reactions, can be dangerous as a possible source of radicals, and, consequently, iron concentrations are tightly regulated by complex homeostatic mechanisms [4]. For this reason, iron is not found in the state of free ions, such as calcium and magnesium, but is always bound to proteins. Generally, iron is present in the trivalent state in the storage molecule ferritin and during transport and in the bivalent state when it performs its biochemical functions [4]. Once transported into the cell, iron can be used as a cofactor for many enzymes, for the assembly of heme, or it can be used to form Fe–S clusters. Excess iron is stored in an inorganic form complexed with phosphate in specific storage molecules, such as ferritins in multicellular organisms and bacterioferritins in bacteria, which differ from ferritins in the presence of a heme molecule [5].

In addition, photosynthetic organisms, including cyanobacteria, have particularly high iron requirements due to Fe-rich photosynthetic machinery. Iron is found within the reaction centers of both photosystems, with 2–3 Fe atoms in photosystem II (PSII) and 12 iron atoms in photosystem I (PSI) [6,7]. Consequently, in cyanobacteria, the intracellular iron concentrations are 4–6 orders of magnitude higher than those of the aquatic environment in which they grow [8]. However, in these organisms, information on the molecular ligands of iron is scarce. Regarding bioaccessibility and bioavailability, it has been well recognized that chemical speciation is more important than the absolute metal concentration [9]. The study of iron speciation could be addressed by hyphenated techniques, including chromatographic fractionation of metal-binding molecules associated with sensitive metal detection in the fractions obtained by atomic absorption spectrometry (AAS) [10].

This research is devoted to study the effects of varying iron concentrations in the growth media on the biochemical composition and the iron bioaccumulation and speciation in *Arthrospira platensis* F&M-C256. Zarrouk medium containing 25 mg L^−1^ of FeSO_4_·7H_2_O was considered as the reference. Two other concentrations of FeSO_4_·7H_2_O were chosen, namely 5 mg L^−1^ and 50 mg L^−1^, to explore the possibility of obtaining biomasses with low and high iron content, respectively. Particular attention is paid to the relationship between iron speciation and the most abundant proteins. Finally, the possible contribution to human nutritional requirements of iron obtained from *A. platensis* F&M-C256 is discussed.

## 2. Results

### 2.1. Production of Arthrospira Platensis F&M-C256 Biomass and Biochemical Composition

**Figure 1:**
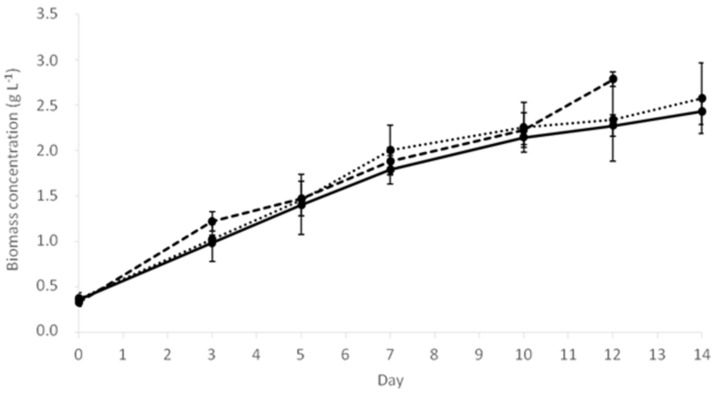
Growth curves of *A. platensis* F&M-C256 grown at three different iron concentrations: 1 mg L^−1^ (continuous line), 5 mg L^−1^ (line with dots), and 10 mg L^−1^ (line with dashes). Values are expressed in g L^−1^ and are reported as means ± SD (*n* = 2).

Figure 1 shows the growth curves of *A. platensis* F&M-C256 cultivated at three different iron concentrations: 1, 5, and 10 mg L^−1^. The biomass concentration at day 0 was set between 0.3 and 0.4 g L^−1^ in all the cultures. Overall, no statistical difference in growth among the cultures grown at different iron concentrations was observed. *A. platensis* F&M-C256 cultures grown at different iron concentrations showed no morphological difference in terms of filaments length and shape during cultivation.

No statistical difference for carotenoids (0.09% dry biomass weight on average) and phenolic content (21.6 mg GAE g^−1^ on average) among *A. platensis* F&M-C256 biomasses grown at different iron concentrations was observed (Table 1). Statistically significant differences were found for chlorophyll *a*, phycocyanin, and antioxidant activity. Chlorophyll *a* content followed the iron concentration in the culture medium with the highest value of 0.9% at the highest iron concentration. Biomass grown at 5 mg L^−1^ of iron contained the highest phycocyanin content (7.8%). Concerning the antioxidant activity, the biomass grown at the lowest iron concentration (1 mg L^−1^) showed the strongest radical scavenging capacity (82.3%).

### 2.2. Iron Bioaccumulation

**Figure 2:**
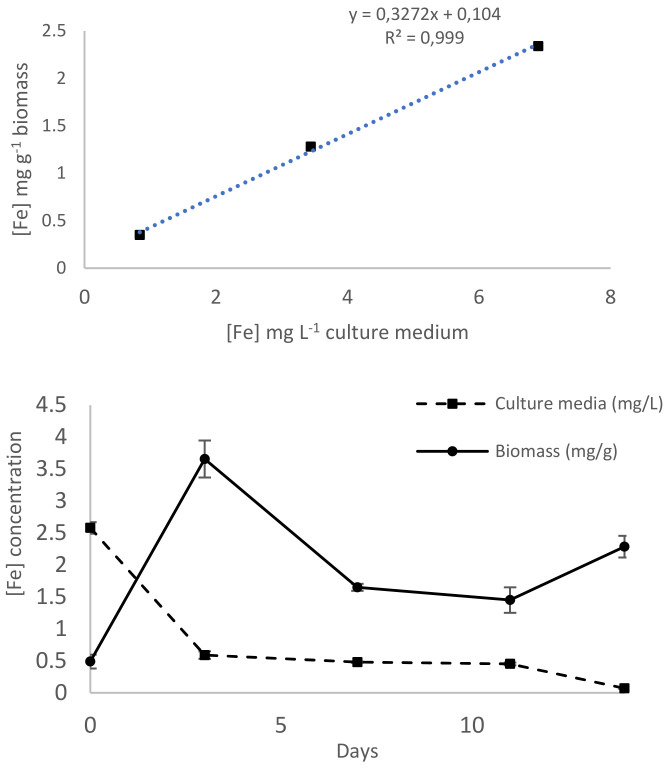
(**Top**) Iron accumulation in algal biomass as a function of iron concentration in the culture medium. (**Bottom**) Iron concentration in culture medium (dotted line reported as mg L^−1^) and iron content in algal biomass (solid line reported as mg g^−1^ dry wt) of cultures with an initial iron concentration of 5 mg L^−1^ during a period of 14 days. Data are reported as mean±SD (*n* = 3).

Iron concentrations in culture media at time zero and in biomasses at the end of cultivation are reported in Table 1. The concentrations measured in culture media were lower than the corresponding nominal concentration. Iron content in the algal biomass varied from 0.35 to 2.34 mg g^−1^ dw and presented a positive and significant correlation with the metal concentrations measured in the culture media (r = 0.999, *p* < 0.05) (Figure 2). To better understand the dynamics of iron accumulation, the metal concentration was also determined at defined time intervals (0, 3, 7, 11, and 14 days) in culture medium and algal biomass (Figure 2) of cultures with an initial iron concentration of 5 mg L^−1^. On day 3, an increase of iron content in algal biomass corresponded to a concomitant decrease in the metal in the culture medium. In the following days (7, 11, and 14), iron content in the biomass varied between 1.5 and 2.5 mg g^−1^. On the same days, decreasing iron concentrations in the culture medium were observed, up to a complete depletion of iron on the last day of the trial, in accordance with an increase in the biomass.

### 2.3. Size Exclusion Chromatography (SEC) and Iron Speciation

The iron distribution between the pellet and the soluble fraction obtained after centrifugation of the algal samples was determined and is reported in Table 1. In all the samples analyzed, iron was more concentrated in the pellets than in the soluble extracts, and the iron percentage in the pellets significantly increased with increasing iron in the culture media. The percentage of iron in the soluble fraction decreased from 10.5% in the biomass grown at 1 mg Fe L^−1^ to 6.04% in the biomass grown at 10 mg Fe L^−1^.

**Figure 3:**
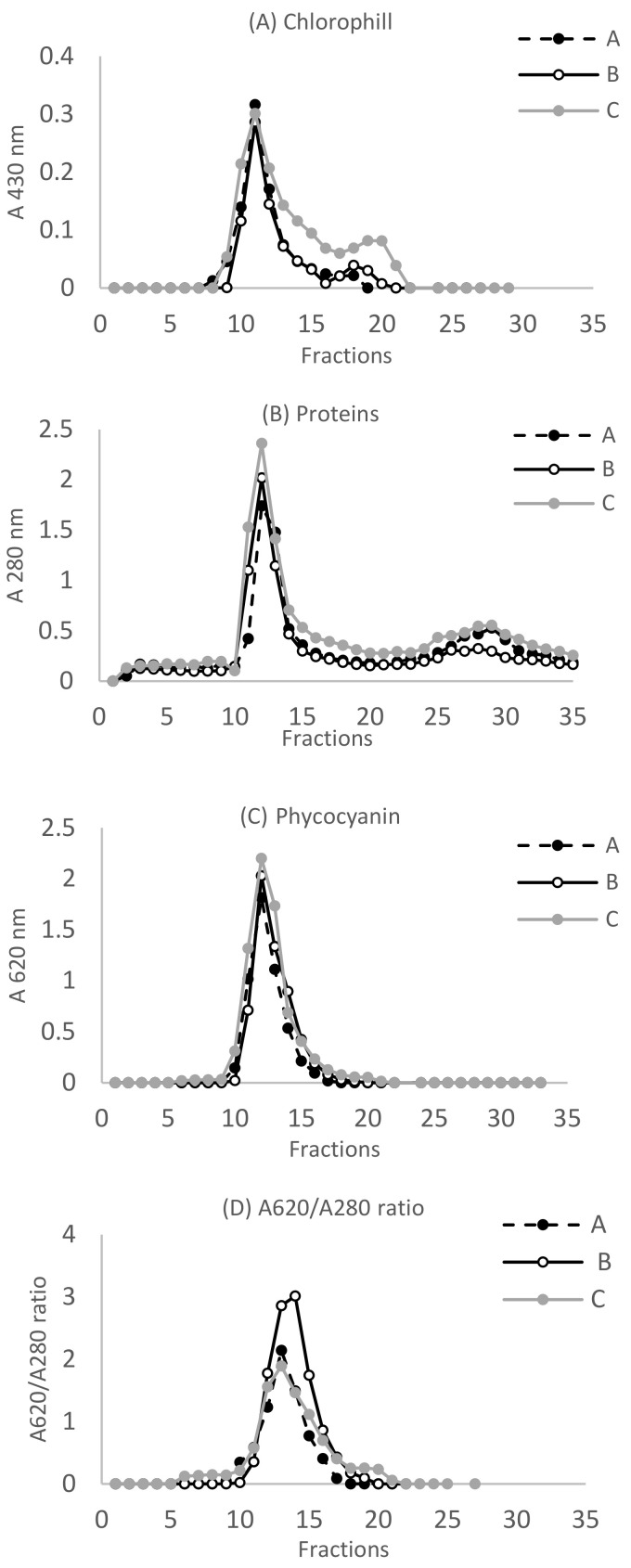
Chromatographic pattern after size exclusion chromatography of extracts A, B, and C obtained from samples grown at 1, 5, and 10 mg Fe L^−1^, respectively. (**A**) Chlorophyll *a* was detected at 430 nm; (**B**) total proteins were measured at 280 nm; (**C**) phycocyanin was measured at 620 nm; (**D**) the purity of phycocyanin was determined by the ratio absorbance at 620 nm/absorbance at 280 nm. Each chromatogram is the mean of two independent chromatographies.

Chlorophyll *a*, total proteins, phycocyanin, and A620/A280 ratio in fractions obtained from SEC are reported in Figure 3A–D. The bulk of proteins eluted in fractions of molecular mass >75 kDa (highest absorbance at 280 nm in fraction 11), while fractions over 22 no longer contained proteins but only small peptides and free amino acids. The absorbance of soluble proteins in fraction 11 and that of phycocyanin in fraction 12 followed this decreasing order: extract C> extract B> extract A (Figure 3B andC). The maximum absorbance of phycocyanin was detected in fraction 12, while the maximum A620/A280 ratio was in fraction 13 for extracts A and C and in fraction 14 for extract B (Figure 3C andD).

**Figure 4:**
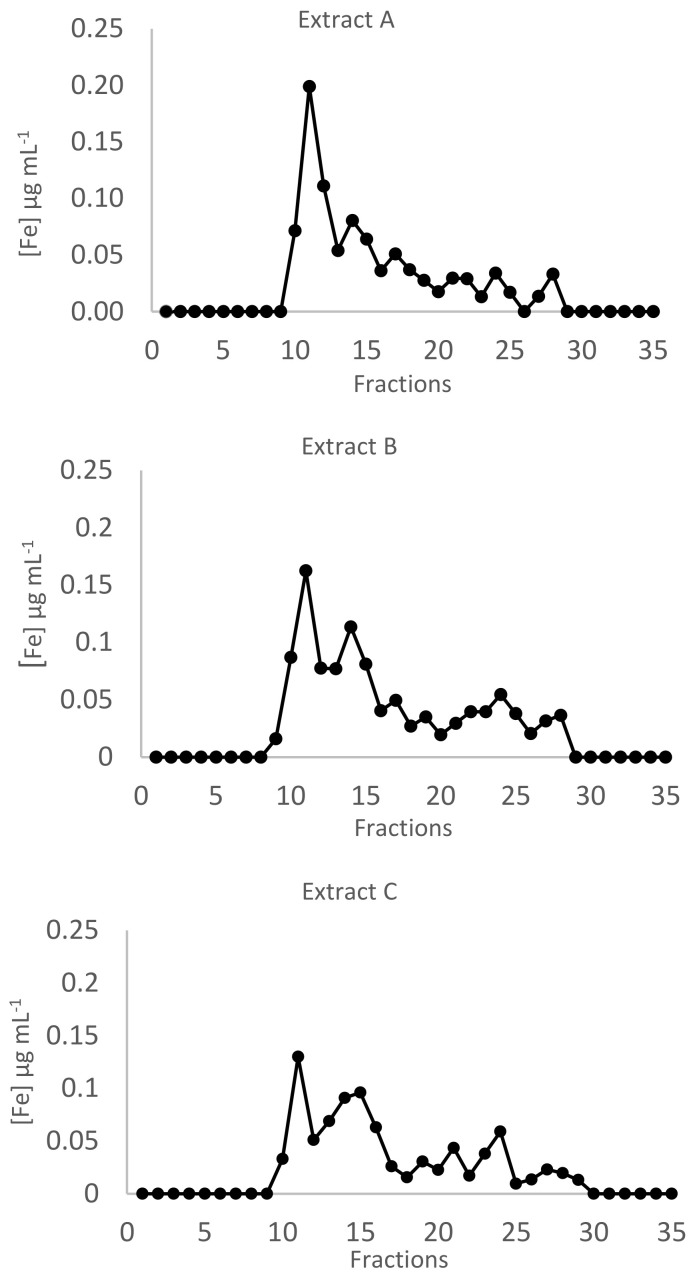
Iron chromatographic profiles after size exclusion chromatography of extracts **A**–**C** obtained from samples grown at 1, 5, and 10 mg Fe L^−1^, respectively. Each chromatogram is the mean of two independent chromatographies. Iron concentration is expressed as µg mL^−1^.

Iron profiles after SEC are reported in Figure 4. Two main peaks were present, namely in fractions 10–12 and in fractions 13–16. However, iron is distributed differently between these two peaks depending on the concentration of the metal in the culture medium. In extract A, iron was mainly bound to ligands with molecular mass >75 kDa (high molecular mass, HMM,) (fractions 10–12). Differently, in extract C, a consistent percentage of the metal (37%) was bound to ligands with intermediate molecular mass (IMM) (fractions 13–16). In all the samples analyzed, low concentrations of iron were bound to ligands with low molecular mass (LMM) (fractions 17–25). An intermediate pattern was present in extract B.

### 2.4. SDS-PAGE and Protein Identification Using Mass Spectrometry

**Figure 5:**
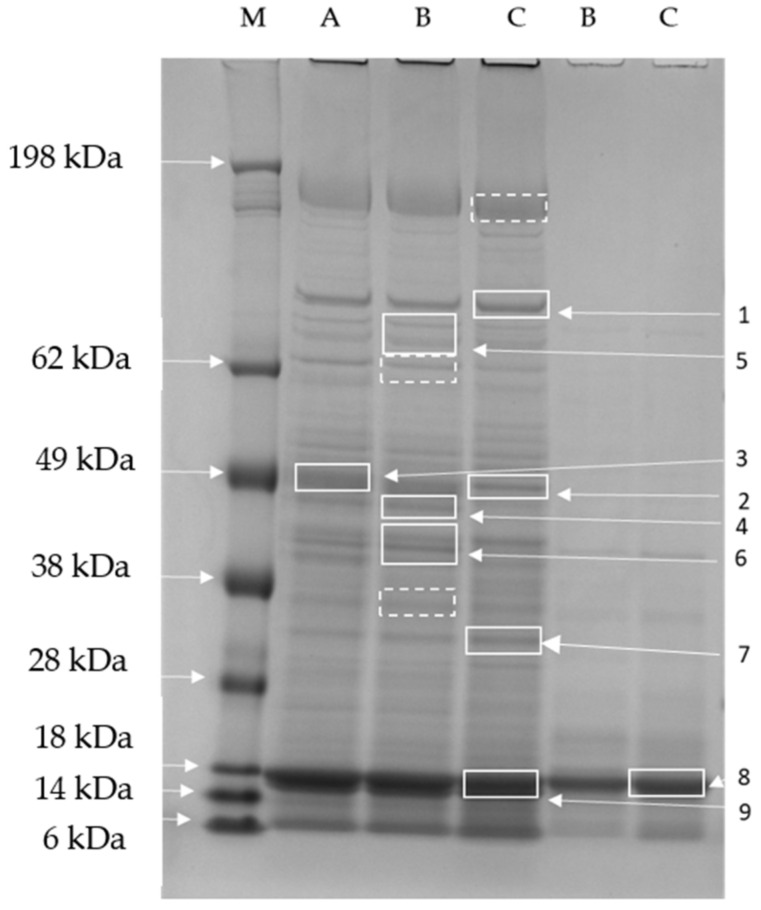
Representative SDS-PAGE (4–12%, Coomassie staining) of fractions obtained after size exclusion chromatography of cellular extracts of samples A, B, and C. Right: Lane 1, molecular mass marker (M) (kDa); lanes 2–4 fraction 11 from samples A (A), B (B), and C (C), respectively; lanes 5–6, fraction 14 from samples B (B) and C (C), respectively. Rectangles indicate bands excised and analyzed by mass spectrometry: continuous line rectangles indicate bands that provided protein identifications (Table 2), while dotted line rectangles indicate bands excised and analyzed by mass spectrometry that did not result in protein identification.

**Figure 6:**
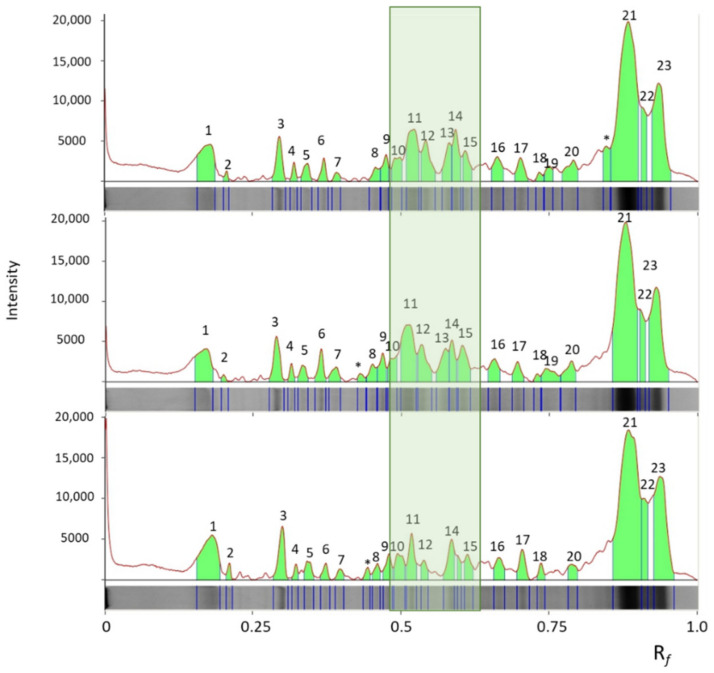
Pherograms obtained from the SDS-PAGE gel reported in Figure 5: lanes 2 (fraction 11, sample A), lane 3 (fraction 11, sample B), and lane 4 (fraction 11, sample C) from the top to the bottom of the figure. In the rectangle, the central zone of the pherogram, containing the excised bands 2, 3, 4, and 6, is outlined, highlighting the different intensities of the protein bands. In the three pherograms, the same peaks are marked by the same number. The peaks present only in one or two pherograms are marked with an asterisk. R_f_ is the migration distance of the protein band through the gel.

The fractions containing the most relevant metal burden, namely fractions 11 and 14, underwent SDS-PAGE on 4–12% gels. A representative gel is reported in Figure 5. Fractions 11, which had the highest absorbance at 280 nm and the highest iron concentration, showed many different protein bands, with apparent molecular mass ranging from 150 to <14 kDa. Fractions 11 from extracts A, B, and C showed quite similar pherograms, with the sole exception of the central zone of the gel in extract C (Figure 6). Fractions 14 showed a major band with an apparent MM of 17–18 kDa.

The most represented bands were cut from the gel and further processed for LC-ESI-QO-MS/MS analysis. Ten proteins were successfully identified from the 12 bands excised from the gel (Table 2). The identity of proteins contained in three bands (Figure 5) could not be found, probably because the protein concentration in the bands was very low, hampering the identification. The biological processes and molecular functions related to these proteins are reported according to gene ontology (GO) and UniProt in Table 2. The proteins identified were involved in relevant biological processes, including energy metabolism (glycolysis), photosynthesis, sulfate assimilation, one-carbon metabolism, carbohydrate transport, and cell redox homeostasis.

## 3. Discussion

### 3.1. Production of A. platensis F&M-C256 Biomass and Biochemical Composition

Iron is a trace element essential for the survival of all organisms, including cyanobacteria. In the present study, no statistical difference in growth among *A. platensis* F&M-C256 cultivated at different iron concentrations was observed, indicating that the lowest iron concentration is able to satisfy the requirements for this essential trace element. Accordingly, Delrue et al. (2017) found that modified Zarrouk’s medium could be diluted up to five times without affecting the growth rates in 28-days batch cultivation [11]. Ismaiel et al. (2018) studied the effects of increasing iron concentration in culture media and reported that concentrations of iron ranging from 0.035 to 0.18 mM (from 1.9 to 10 mg L^-1^) had no significant effect on growth [12].

In photosynthetic organisms, iron is essential for chlorophyll biosynthesis. Accordingly, in the present research, a significant increase in chlorophyll *a* was found in *A. platensis* F&M-C256 with the increase of iron concentration in the growth medium. Ismaiel et al. (2018) found a similar effect in *A. platensis* for chlorophyll *a* and phycocyanin [12]. In our research, biomass grown at the standard iron concentration of Zarrouk medium (5 mg L^−1^) contained the highest phycocyanin content (about 8%). Xing et al. (2007) found that iron limitation induces phycobiliprotein degradation in cyanobacteria, which could explain the lower phycocyanin amount found for *A. platensis* F&M-C256 grown at 1 mg Fe L^−1^ [13]. In our study, as the iron concentration increased, a reduction in antioxidant capacity was observed. It is worth pointing out that in the sample of *A. platensis* F&M-C256 grown at the highest iron concentration, the bands containing glutathione oxidoreductase and adenosylhomocysteinase, two antioxidant enzymes, are actually less intense (bands 4 and 6, Figure 5 and Figure 6), suggesting lower amounts of these proteins and less effective antioxidant defenses.

### 3.2. Iron Bioaccumulation

The data obtained in the present research showed iron concentrations in the culture media lower than the nominal ones. The fate of iron in the alkaline medium used for *A. platensis* cultures is still poorly explored. It has been hypothesized that iron precipitates as FePO_4,_ determining the decrease in the metal concentration available for cell growth [14]. Moreover, it has been reported by Perfiliev et al. (2018) that iron concentrations in the culture medium higher than 5 µg L^−1^ causes sedimentation of the metal, determining a lower bioaccessibility of iron [15]. In the present study, the discrepancy between the iron nominal concentration and the concentration determined in culture media by AAS suggests iron precipitation.

Iron concentrations measured in *A. platensis* F&M-C256 biomass varied from 352 to 2342 µg g^−1^ dw. These data are in accordance with concentrations from 300 to 2160 µg g^−1^ reported in the literature for iron in different *A. platensis* samples [16,17]. It is known that the wide variability of iron concentrations in *Arthrospira* is due to the metal bioavailability in the water, which, in turn, is dependent upon environmental parameters, such as salinity, pH, light intensity, and nutrients [18].

The ability of cyanobacteria to bioaccumulate metals is an interesting opportunity to obtain biomasses enriched with specific trace elements. This behavior can be exploited in different ways, from bioremediation of polluted environments to the production of high-value food supplements. On average, for biomass obtained at the highest iron concentration in this research, daily supplementation with the commonly recommended dietary intake of 3.0 g of spirulina would constitute 64% of the population reference intake (PRI) of 11 mg day^−1^ reported by EFSA for iron [19]. It has been reported that *A. platensis* trichomes accumulate iron mostly in the form of Fe^3+^ as ferrihydrite [15]. Therefore, the bulk of iron present in the pellet of all the examined samples should be stored in this inorganic form. The chemical form of iron, in particular, the presence of heme or non-heme iron, is critical in determining the bioavailability of this essential element. Puyfoulhoux et al. (2001) compared the iron bioavailability of iron-fortified spirulina with different iron sources by measuring ferritin formation in Caco-2 cells exposed to digests obtained from meat, yeast, and wheat [20]. The results obtained by these authors with spirulina were indicative of a metal bioavailability not significantly different from that exhibited by the meat digest, despite the different chemical forms of iron. Moreover, the effect of spirulina supplementation has been tested on anemia in senior citizens. Over the 12-weeks study period, an increase in mean corpuscular hemoglobin was detected in both sexes [21], indicating a possible application as a high-value supplement to improve anemia. However, the biochemical basis of this effect is still to be discovered, and future studies should be focused on large clinical trials.

On the other hand, the biomass with the lowest iron concentration associated with a higher antioxidant activity could be an interesting supplement for people subjected to a reduced iron dietary intake due to cardiovascular risk. In fact, it is well recognized that high iron intake and stores are associated with an increased risk of iron-induced oxidative stress, inflammation, and cardiovascular diseases [22].

### 3.3. Iron Speciation and Protein Identification

In addition to inorganic iron, a percentage of the metal varying from 6% (extract C) to 10% (extract A) was present in the soluble fraction, likely bound to proteins. Studies on trace element speciation, i.e., the distribution of the element among defined chemical species in biological systems, are rare in cyanobacteria. In this research, non-denaturing SEC followed by iron analysis of the chromatographic fractions using AAS allowed the separation of soluble proteins. To further fractionate these proteins, before identification with mass spectrometry, an additional separation step using SDS-PAGE electrophoresis was applied to chromatographic fractions 11 and 14, which contained the highest concentrations of iron.

A total of 3 of the 10 proteins identified by mass spectrometry are at the crossroad between photosynthesis and iron metabolism. In all the samples, the most abundant protein in fractions 11 and 14 identified in bands 8 and 9 is C-phycocyanin (C-PC), a multimeric blue protein belonging to the phycobiliprotein family. In cyanobacteria, C-PC is an essential pigment of the phycobilisome and contains a prosthetic group similar to bilverdin and bilirubin, known as phycocyanobilin (PCB). This protein consists of two subunits, which bind one and two PCB, respectively. The molecular mass (MM) of α and β subunits varies depending on the species, from 11 to 24.4 [23]. The MM obtained in this study for the α subunit is like that reported for spirulina by Patel et al. (2005) [24]. In addition to its essential function as a light-capturing antenna for photosystem II, C-PC is a potent scavenger of hydroxyl and peroxyl radicals and can bind iron in vitro [25]. Bermejo et al. concluded that the antioxidant capacity of *Spirulina platensis* protein extract arises from both radical scavenging and Fe^2+^ chelation activity. Other evidence was previously reported by Bhat and Madyastha (2000) [26], who found that phycocyanin interacts with iron with an association constant of 1.11 ± 0.06 × 10^5^ M, and recently by Kim et al. (2014), who purified an iron-chelating peptide from spirulina protein hydrolysates [27]. In our study, the abundance of C-PC in fractions 11 and 14, which contain the bulk of iron, adds evidence to the role of C-PC as a potential iron-binding protein also in vivo. 

One other protein related to photosynthesis is ferredoxin NADP(H) oxidoreductase (EC 1.18.1.2) (FNR), identified in band 8 [28]. This enzyme receives electrons from ferredoxin and reduces NADP+ to NADPH [29]. Therefore, its main function is to ensure the final step of photosynthetic electron transport, providing NADPH for CO_2_ assimilation. In cyanobacteria, FNR has been found to be associated mostly with phycobilisomes, and two isoforms have been reported: a short isoform of 33 kDa, similar to plant stromal FNR, and a long isoform of 45 kDa [29]. The FNR identified in this study had a molecular weight of 34 kDa, suggesting the presence of the short isoform in *A. platensis*. The concomitant identification of C-PC and FNR in fraction 11 is suggestive of the presence of phycobilisomes, if not intact in their complex molecular architecture, at least as sub-complexes, including some of the proteins that take part in the structure. It is important to point out that the extracts prepared in this study are obtained in non-denaturing conditions, which preserve the functional structure of proteins and protein complexes. However, during the subsequent step of SDS-PAGE, denaturing conditions are applied, resulting in protein denaturation, including dissociation of protein subunits, which appear after electrophoresis with MM lower than the active polymers.

Iron-uptake porin identified in band 2 is another interesting protein involved in iron metabolism. Cyanobacteria require a large amount of iron to maintain the photosynthetic machinery. Consequently, iron availability is a key factor in controlling cyanobacteria populations and frequently is a limiting factor for biomass growth. In *Synechocystis* sp, complex membrane pathways are present to cope with the intake of iron, including the presence of the TonB-dependent transport system, which transports the ferri-siderophore complexes, especially in the situation of iron depletion [30]. However, siderophore-mediated iron uptake is functioning at a low rate in iron-replete environments, suggesting the existence of alternative systems for iron uptake. Recently, Qiu et al. (2021) reported the presence of a specific iron-selective porin in *Synechocystis* sp [6]. The identification of this protein in the extracts of the samples analyzed in this research adds in vivo evidence of the presence of this protein also in *A. platensis*. Interestingly, the intensity of this band decreases as the iron concentration in the culture medium increases (Figure 5 and Figure 6), suggesting the existence of a regulatory mechanism controlling intracellular iron content.

Iron challenge followed by increased intracellular metal concentrations exposes organisms to an increased risk of oxidative stress. Two of the identified proteins have recognized antioxidant activity: glutathione oxidoreductase identified in band 4 and adenosyl-homocysteinase identified in band 6. Particularly, the first enzyme is a key player in the antioxidant defense system by reducing glutathione (GSH) from its oxidized form (GSSG) [31]. The MM of 47 kDa determined in this study for the subunit of glutathione oxidoreductase is confirmatory of that previously reported by Rendón et al. (1986) [32].

Three of the identified proteins are involved in carbohydrate transport and metabolism. Phosphoglycerate kinase (PGK), identified in band 6, is an essential enzyme in all living organisms ensuring the production of one of the two ATP resulting from anaerobic glycolysis. Moreover, in photosynthetic organisms, PGK is involved in the Calvin-Benson-Bassham (CBB) cycle catalyzing the phosphorylation of 3-phosphoglycerate to 1,3-biphosphoglycerate as part of the reactions that regenerate ribulose-1,5-bisphosphate. Transketolase, identified in band 5, is also involved in the CBB cycle and plays an important role in connecting the CBB to the pentose pathway. This enzyme has been previously identified in *A. platensis* by Wang et al. (2013) [33]. Finally, a carbohydrate-selectiveporin (OprB family) was identified in band 3. This protein is a component of the carbohydrate transport system, regulating the binding and transmembrane flux of several carbohydrates.

## 4. Materials and Methods

### 4.1. Production of Arthrospira platensis F&M-C256 Biomass

*A. platensis* F&M-C256 belongs to the Fotosintetica & Microbiologica S.r.l. Microalgae Culture Collection (Sesto Fiorentino, Italy). The cyanobacterium was grown, in duplicate cultures, in 300 mL bubble tubes in a batch regime, at 25 °C and 150 µmol photons m^−2^ s^−1^. The air: CO_2_ mixture was injected at a flow rate of 0.3 L L^−1^ min^−1^ after filtration through 0.2 μm sterile PTFE membranes (Sartorius Stedim Biotech GmbH, Göttingen, Germany). The pH was monitored daily and kept constant at about 9.3 by regulating the CO2 supply.

Zarrouk medium at three different iron concentrations was prepared. Iron was added to the culture medium as FeSO_4_.7H_2_O at initial concentrations of 5, 25 (as in the Zarrouk medium recipe), and 50 mg L^−1^, corresponding to nominal iron concentrations of 1, 5, and 10 mg L^−1^. *A. platensis* F&M-C256 cultures were harvested by centrifugation after 12 or 14 days of cultivation. The biomasses were then frozen, lyophilized, powdered, and stored at −20 °C until use. A second batch at 5 mg L^−1^ of iron was performed to evaluate iron accumulation in the biomass and iron depletion in the medium over time by sampling the culture every three or four days. The samples were centrifuged at 3600 g for 15 min. The supernatant and the pellet were separately recovered and frozen. The pellet was lyophilized and powdered. Both the supernatants and the pellets were stored at −20 °C until use.

Microscopic observations of the cultures were carried out daily using a direct-light microscope (Eclipse E 200, Nikon, Tokyo, Japan). Cultures growth was estimated daily by measurement of dry weight concentration.

### 4.2. Pigments, Total Phenolic, and DPPH Analyses

*A. platensis* F&M-C256 biomasses obtained at the end of the cultivation period at the three iron concentrations were analyzed for phycocyanin, chlorophyll *a*, and total carotenoids. Phycocyanin was extracted with CaCl_2_ according to Herrera et al. (1989), and its concentration was determined spectrophotometrically (Cary 50, Varian, Palo Alto, CA USA) using Bennet and Bogorad (1973) equations [34,35]. Chlorophyll *a* and total carotenoids were extracted in 90% acetone in water and determined spectrophotometrically (Cary 60 UV-Vis, Agilent Technologies, Santa Clara, CA, USA) according to Jeffrey and Humphrey (1975) and Parson and Strickland (1963), respectively [36,37]. To evaluate the radical scavenging capacity, the 2,2-diphenyl-1-picrylhydrazyl (DPPH) radical scavenging assay was carried out according to Rajauria et al., 2013) [38], and total phenolic content was determined using the Folin–Ciocalteu assay [39].

### 4.3. Metal Analysis

To avoid contamination, all the reagents were handled carefully; polyethylene disposables were thoroughly washed with HCl 1 N under a fume hood, and disposable gloves were worn during the procedure. All the reagents were from Merck (Merk, Darmstadt, Germany); the acids were of Suprapur grade. Samples of lyophilized biomasses (400 mg) and pellets obtained as described in Section 4 (200 mg) were placed in individual acid-washed Teflon jars and were digested with 1–2 mL 65% HNO_3_ and 0.25–0.5 mL 30% H_2_O_2_ in a microwave oven for 5 min at 250 W, 5 min at 400 W, 5 min at 500 W and 1 min at 600 W. The cooled samples were transferred into 5–10 mL polyethylene volumetric flasks and were directly analyzed using a flame atomic spectrophotometer equipped with a deuterium lamp background correction (AAnalyst 100, Perkin Elmer, Waltham, MA, USA) for iron determination. Supernatants obtained as described in Section 4.4 were directly analyzed without any further treatment. All the samples were run in batches, which included blanks; there was no evidence of any contamination in these blanks. The accuracy of the method was evaluated using the analysis of international standards (ERM^®^-BB422 fish muscle). The concentrations found with the method used in this study fell into the certified uncertainty interval given by ERM, corresponding to a 95% confidence level. The iron detection limit for flame atomic spectrophotometry was 0.04 μg mL^−1^. Iron concentrations were reported as μg mL^−1^ or μg g^−1^ depending on the sample analyzed.

### 4.4. Iron Speciation and Size Exclusion Chromatography (SEC)

Two hundred milligrams of lyophilized biomass from *A. platensis* cultivated in the presence of nominal Fe concentrations of 1, 5, and 10 mg L^−1^ were crushed in a mortar with a pestle under liquid nitrogen and homogenized in 3 volumes (*v/w*) of Tris–HCl 20 mM and 10 mM mercaptoethanol, pH 8.6, using an Ultraturrax (IKA, Staufen, Germany) homogenizer. The homogenate was sonicated for 10 min at 38 kHz and then centrifuged at 24,000× *g* for 40 min at 4 °C, obtaining the separation between supernatant and pellet. The supernatant obtained from *A. platensis* cultivated in the presence of nominal iron concentrations of 1 mg L^−1^ is indicated as extract A, while the supernatants obtained in the presence of nominal iron concentrations of 5 and 10 mg L^−1^ are indicated as extract B and C, respectively. For each extract, a volume of 0.8 mL supernatant was applied to a Sephadex G-75 chromatography column (0.9 × 90 cm). The column was calibrated using a commercial kit (GF70-1KT, Sigma-Aldrich, St Louis, MO, USA). Fractions of 1.5 mL were collected and analyzed for iron concentration using the direct aspiration of the solution into the flame of an atomic absorption spectrophotometer as described above. The pellets obtained from the centrifugation of the homogenate were digested in a microwave oven, as reported above, and iron concentration was determined using a flame atomic absorption spectrophotometer (AAnalyst 100, Perkin Elmer, Waltham, MA, USA) as reported in “Metal analysis”. In the supernatants and fractions obtained from size exclusion chromatography, total proteins, phycocyanin, and chlorophyll were determined by direct measurement of the absorbance at 280, 615, and 430 nm, respectively (DeNovix DS-11 Series Spectrophotometer, Wilmington, DE, USA). The purity of phycocyanin was determined by the ratio absorbance at 620/absorbance at 280.

### 4.5. SDS-PAGE

Five µg of protein were loaded onto 4–12% Bis-Tris polyacrylamide gels (NuPage/Thermo Fisher Scientific, Waltham, MA, USA), and electrophoresis (PAGE) was carried out in an Xcell SureLock Mini-Cell with 2-(N-morpholino) ethanesulfonic acid buffer (MES; NuPage/Thermo Fisher Scientific, Waltham, Massachusetts, MA, USA), containing sodium dodecyl sulfate (SDS). Each gel was also loaded with standard proteins of known molecular weight (SeeBlue™ Plus2 Pre-stained Protein Standard, Thermo Fisher Scientific, Waltham, MA, USA). The electrophoresis system was connected to a power supply (Power Pack Basic—Bio-Rad, Hercules, CA, USA) with a constant voltage of 200 V for 40 min. The gels were stained with Coomassie G250 compatible with mass spectrometry analysis. After staining, each gel was digitalized by ChemiDocMP (BioRad, Hercules, CA, USA), and pherograms were obtained using ImageLab 5.2.1 software (BioRad, Hercules, CA, USA).

### 4.6. Protein Identification Using Mass Spectrometry

The most represented bands were manually cut from the gel and underwent “in-gel” tryptic digestion. The bands were first destained with acetonitrile (ACN); the proteins were then reduced by 10 mM Dithiothreitol (DTT) at 56 °C for 30 min and were subsequently alkylated using 55 mM iodoacetamide for 20 min in the dark. After drying, the proteins were digested with 12.5 ng µL^−1^ Trypsin (Promega, Madison, WI, USA) by incubation overnight at 37 °C. Subsequently, the peptides were extracted using a solution composed of 1% trifluoroacetic acid/50% ACN and were finally concentrated in a vacuum dryer (Eppendorf Concentrator Plus).

The dried digested samples were resuspended in 95% water/3% ACN/2% formic acid and analyzed using a UHPLC-MS QExactive™ system (Thermo Fisher Scientific, Reinach, Switzerland), composed of a UHPLC 3000 Ultimate System coupled to an ESI-QExactive™ Hybrid Quadrupole-Orbitrap™ mass spectrometer (LC-ESI-QO-MS/MS System). The separations were carried out on a ZORBAX RRHD Eclipse Plus C18 column (50 × 2.1 mm ID, 1.8 μm particle size, Agilent Technologies, Santa Clara, CA, USA) with a mobile phase composed of 0.1% aqueous formic acid solution (A) and ACN (B), using the following gradient elution at a flow rate of 0.3 mL/min: 0 to 3 min: isocratic at 2% (B); 3 to 21 min: linear gradient from 2% to 27% (B); 21 to 25 min: linear gradient from 27% to 90% (B); 25 to 28 min: isocratic at 90% (B); 28 to 28.1 min: linear gradient from 90% to 2% (B). An equilibration period of 6.9 min was interposed between each run. Nitrogen was used for spray stabilization, for collision-induced dissociation experiments in the higher energy collision dissociation (HCD) cell, and as damping gas in the C-trap. The ESI source operated in positive ionization mode, with a capillary voltage of 3.5 kV. The analyses were checked using Xcalibur™ software (version 29 build 2926).

The raw mass data acquired, converted into Mascot generic format using MsConvert (version 3.0.10730, ProteoWizard tools), were searched using the MASCOT search engine (version 2.7, http://mascot.cigs.unimo.it/mascot, accessed on 30 May 2022). Trembl and SwissProt, together with C-RAP protein databases, were selected for peptide sequence and contaminant searching, respectively, setting the following restriction parameters: trypsin as a proteolytic enzyme, max two missed cleavages; peptide mass tolerance ± 10 ppm (for precursor ions) and fragment mass tolerance ± 0.02 Da (for product ions); unrestricted protein mass; carbamidomethylation to cysteine residues as fixed modification, and deamidated (NQ) and oxidated (M) methionine as variable modifications.

### 4.7. Statistical Analysis

Part of the statistical analysis was carried out using statistical software (RStudio-1.2.1335 Statistical and R, R v3.4.3, Development Core Team, Vienna, Austria) and part using Statgraphics Centurion XV (StatPoint Technologies Inc., Washington, DC, USA). Statistical differences between values of chlorophyll a, carotenoids, phycocyanin, antioxidant activity, and phenolic content were determined using ANOVA followed by Duncan’s multiple range tests (MRT) to determine the least significant difference (LSD). All data were evaluated using standard descriptive statistics and reported as mean ± standard deviation (SD). A *p*-value < 0.05 was considered significant.

## 5. Conclusions

This research explored the possibility of obtaining cyanobacterial biomasses with different iron concentrations by varying the metal concentration in the culture media. *A. platensis* F&M-C256 was demonstrated to be a suitable model organism for the above purpose. The study of iron speciation using hyphenated techniques represents an innovation opening new scenarios in the field of iron metabolism in cyanobacteria. The abundance of C-PC in fractions 11 and 14, which contain the bulk of soluble iron, adds evidence to the role of C-PC as an Fe-binding protein in vivo. Finally, the iron concentrations measured in the three *A. platensis* F&M-C256 biomasses (from 0.35 to 2.34 mg Fe g^−1^ dry weight) can be considered of interest to produce spirulina-based supplements with low and high iron content to satisfy different groups of consumers.

## Figures and Tables

**Table 1:** Iron concentration in the culture medium at time zero and chlorophyll *a*, carotenoids, phycocyanin, antioxidant activity, phenolic content, iron concentration in the biomass, algal pellet, and cytosolic extract at the end of cultivation of *A. platensis* F&M-C256 are reported for each iron nominal concentration of 1, 5, and 10 mg L^−1^. Values are reported as means ± SD (*n* = 3). Different letters in a column indicate a significant difference (*p* < 0.05).

[Fe] Nominal Concentration (mg L^−1^)	[Fe] Culture Medium at Time Zero (mg L^−1^)	Chlorophyll *a* (% d.w.)	Carotenoids (% d.w.)	Phycocyanin (% d.w.)	Antioxidant Activity (% RSA)	Phenolics (mg GAE g^−1^)	[Fe] Biomass (mg g^−1^ dw)	[Fe] Algal Pellet (mg g^−1^ dw)	[Fe] Algal Cytosolic Extract (µg mL^−1^)
[Fe] 1	0.84 ^c^ ± 0.04	0.50 ^c^ ± 0.05	0.08 ^a^ ± 0.03	5.16 ^c^ ± 0.14	82.29 ^a^ ± 0.57	23.93 ^a^ ± 1.22	0.35 ^c^ ± 0.02	0.056 ^c^ ± 0.003	1.53 ^b^ ± 0.11
[Fe] 5	3.44 ^b^ ± 0.18	0.66 ^b^ ± 0.10	0.08 ^a^ ± 0.01	7.81 ^a^ ± 0.48	73.83 ^b^ ± 1.57	20.26 ^a^ ± 0.77	1.28 ^b^ ± 0.03	0.160 ^b^ ± 0.020	3.68 ^a^ ± 0.42
[Fe] 10	6.90 ^a^ ± 0.42	0.90 ^a^ ± 0.16	0.11 ^a^ ± 0.04	6.10 ^b^ ± 0.90	72.69 ^b^ ± 0.57	20.49 ^a^ ± 1.14	2.34 ^a^ ± 0.10	0.248 ^a^ ± 0.021	4.23 ^a^ ± 0.16

dw: dry weight, RSA: radical scavenging activity, GAE: gallic acid equivalent.

**Table 2:** Protein identification in fractions from cytosolic extracts of *A. platensis* using mass spectrometry.

Band ^a^	Entry Name ^b^	Protein Full Name	MM (Da) ^c^	Organism	Score ^d^	Sign Pept ^e^	Sign Seq ^f^	Molecular Function	Biological Process
1	A0A015IXE4_ 9GLOM	J-domain containing protein	33,255	*Arthrospira platensis*	21	1	1	DNA binding	
2	WP_035760125.1	Iron-uptake porin	67,484	*Arthrospira platensis*	59	6	6	Porin activity	Carbohydrate transport
3	K1VSN7_ ARTPT	Carbohydrate-selective porin OprB	68,358	*Arthrospira platensis*	119	13	12	Porin activity	Carbohydrate transport
4	H1WGM5_ 9CYAN	Glutathione oxidoreductase	48,615	*Arthrospira platensis*	155	10	8	Oxidoreductase	Cell redox homeostasis—Cell oxidant detox
5	H1WMK8_ 9CYAN	Transketolase	72,899	*Arthrospira platensis*	67	3	2	Transferase, metal ion-binding	Pentose shunt
6	H1W8T2_ 9CYAN	Phosphoglycerate kinase	42,306	*Arthrospira platensis*	213	12	8	Transferase	Glycolysis
	H1WJF3_ 9CYAN	Adenosylhomo cysteinase	46,765	*Arthrospira platensis*	124	7	7	Hydrolase	One-carbon metabolism
	H1WJ30_ 9CYAN	Sulfate adenylyltransferase	44,594	*Arthrospira platensis*	92	6	6	Transferase	Sulfate assimilation
7	H1WF80_ 9CYAN	Ferredoxin NADP reductase	45,396	*Arthrospira platensis*	74	7	6	Oxidoreductase	Photosynthesis
8–9	A0A0C4VZ70_ ARTPT	CpcA protein	17,703	*Arthrospira platensis*	682	34	8	Chromophore	Photosynthesis

^a^ Number of the identified band as marked in Figure 5. ^b^ Protein entry name from the UniProt knowledge database. ^c^ Theoretical protein molecular mass. ^d^ The highest scores obtained with the Mascot search engine. ^e^ Significant peptides: total number of significant peptides matching the identified proteins. ^f^ Significant sequences: total number of significant distinct sequences matching the identified proteins.

## Data Availability

Not applicable.

## References

[1] Lafarga T., Fernández-Sevilla J.M., González-López C., Acién-Fernández F.G. (2020). Spirulina for the food and functional food industries. Food Res. Int..

[2] Niccolai A., Chini Zittelli G., Rodolfi L., Biondi N., Tredici M.R. (2019). Microalgae of interest as food source: Biochemical composition and digestibility. Algal Res..

[3] Kulshreshtha A., Anish J.Z., Jarouliya U., Bhadauriya P., Prasad G.B.K.S., Bisen P.S. (2008). Spirulina in health care management. Curr. Pharm. Biotechnol..

[4] Andrews N.C. (2008). Forging a field: The golden age of iron biology. Blood.

[5] Kranzler C., Rudolf M., Keren N., Schleiff E. (2013). Iron in Cyanobacteria. Adv. Bot. Res..

[6] Qiu G.W., Jiang H.B., Lis H., Li Z.K., Deng B., Shang J.L., Sun C.Y., Keren N., Qiu B.S. (2021). A unique porin meditates iron-selective transport through cyanobacterial outer membranes. Environ. Microbiol..

[7] Raven J.A., Evans M.C.W., Korb R.E. (1999). The role of trace metals in photosynthetic electron transport in O_2_-evolving organisms. Photosyn. Res..

[8] Morel F.M.M., Price N.M. (2003). The Biogeochemical Cycles of Trace Metals in the Oceans. Environ. Sci..

[9] Beck K.L., Conlon C.A., Kruger R., Coad J. (2014). Dietary determinants of and possible solutions to iron deficiency for young women living in industrialized countries: A review. Nutrients.

[10] Mounicou S., Szpunar J., Lobinski R. (2009). Metallomics: The concept and methodology. Chem. Soc. Rev..

[11] Delrue F., Alaux E., Moudjaoui L., Gaignard C., Fleury G., Perilhou A., Richaud P., Petitjean M., Sassi J.F. (2017). Optimization of *Arthrospira platensis* (Spirulina) Growth: From Laboratory Scale to Pilot Scale. Fermentation.

[12] Ismaiel M.M., Piercey-Normore M.D., Rampitsch C. (2018). Proteomic analyses of the cyanobacterium *Arthrospira* (*Spirulina*) *platensis* under iron and salinity stress. Environ. Exp. Bot..

[13] Xing W., Huang W.M., Li D.H., Liu Y.D. (2007). Effect of iron on growth, pigment content, photosystem II efficiency, and siderophores production of *Microcystis aeruginosa* and *Microcystis wesenbergii*. Curr. Microbiol..

[14] Belay A. (1997). Mass Culture of Spirulina Outdoors—The Earthrise Farms Experience (Chapter 8). Spirulina Platensis Arthrospira: Physiology, Cell-Biology and Biotechnology.

[15] Perfiliev Y.D., Tambiev K., Konnychev M.A., Skalny A.V., Lobakova E.S., Kirpichnikov M.P. (2018). Mössbauer spectroscopic study of transformations of iron species by the cyanobacterium *Arthrospira platensis* (formerly *Spirulina platensis*). J. Trace Elem. Med. Biol..

[16] Cepoi L., Chiriac T., Rudi L., Djur S., Zosim L., Bulimaga V., Batir L., Elenciuc D., Rudic V. (2017). Spirulina as a Raw Material for Products Containing Trace Elements (Chapter 19). Recent Advances in Trace Elements.

[17] Rzymski P., Budzulak J., Niedzielski P., Klimaszyk P., Proch J., Kozak L., Poniedziałek B. (2019). Essential and toxic elements in commercial microalgal food supplements. J. Appl. Phycol..

[18] Wild K.J., Steinga H., Rodehutscord M. (2018). Variability in nutrient composition and in vitro crude protein digestibility of 16 microalgae products. J. Anim. Physiol. Anim. Nutr..

[19] European Food Safety Authority (EFSA) (2015). Scientific Opinion on Dietary Reference Values for iron. EFSA J..

[20] Puyfoulhoux G., Rouanet J.M., Besançon P., Baroux B., Baccou J.C., Caporiccio B. (2001). Iron Availability from Iron-Fortified Spirulina by an in Vitro Digestion/Caco-2 Cell Culture Model. J. Agri. Food Chem..

[21] Selmi C., Leung P.S.C., Fischer L., German B., Yang C.Y., Kenny T.P., Cysewski G.R., Gershwin M.E. (2011). The effects of Spirulina on anemia and immune function in senior citizens. Cell. Mol. Immunol..

[22] Hunnicutt J., He K., Xun P. (2014). Dietary iron intake and body iron stores are associated with risk of coronary heart disease in a meta-analysis of prospective cohort studies. J. Nutr..

[23] Fernández-Rojas B., Hernández-Juárez J., Pedraza-Chaverri J. (2014). Nutraceutical properties of phycocyanin. J. Funct. Foods..

[24] Patel A., Mishra S., Pawar R., Ghosh P.K. (2005). Purification and characterization of C-Phycocyanin from cyanobacterial species of marine and freshwater habitat. Protein Expr. Purif..

[25] Bermejo P., Piñero E., Villar Á.M. (2008). Iron-chelating ability and antioxidant properties of phycocyanin isolated from a protean extract of *Spirulina platensis*. Food Chem..

[26] Bhat V.B., Madyastha K.M. (2000). C-Phycocyanin: A potent peroxyl radical scavenger in vivo and in vitro. Biochem. Biophys. Res. Commun..

[27] Kim K., Choi J., Ji Y., Park S., Do H., Hwang C., Lee B., Holzapfel W. (2014). Impact of bubble size on growth and CO_2_ uptake of *Arthrospira* (*Spirulina*) *platensis* KMMCC CY-007. Bioresour. Technol..

[28] Vorphal M.A., Bruna C., Wandersleben T., Dagnino-Leone J., Lobos-González F., Uribe E., Martìnez-Oyanedel J., Bunster M. (2017). Molecular and functional characterization of ferredoxin NADP(H) oxidoreductase from *Gracilaria chilensis* and its complex with ferredoxin. Biol. Res..

[29] Morsy F.M., Nakajima M., Yoshida T., Fujiwara T., Sakamoto T., Wada K. (2008). Subcellular localization of ferredoxin-NADP+ oxidoreductase in phycobilisome retaining oxygenic photosysnthetic organisms. Photosyn. Res..

[30] Qiu G.W., Lou W.J., Sun C.Y., Yang N., Li Z.K., Li D.L., Zang S.S., Fu F.X., Hutchins D.A., Jiang H.B. (2018). Outer membrane iron uptake pathways in the model cyanobacterium *Synechocystis* sp. strain PCC 6803. Appl. Environ. Microbiol..

[31] Sannasimuthu A., Kumaresan V., Anilkumar S., Pasupuleti M., Ganesh M.R., Mala K., Paray B.A., Al-Sadoon M.K., Albeshr M.F., Arockiaraj J. (2019). Design and characterization of a novel *Arthrospira platensis* glutathione oxido-reductase-derived antioxidant peptide GM15 and its potent anti-cancer activity via caspase-9 mediated apoptosis in oral cancer cells. Free Radic. Biol. Med..

[32] Rendón J.L., Calcagno M., Mendoza-Hernández G., Ondarza R.N. (1986). Purification, properties, and oligomeric structure of glutathione reductase from the cyanobacterium *Spirulina maxima*. Arch. Biochem. Biophys..

[33] Wang H., Yang Y., Chen W., Li D., Zhao X., Wang X., Li A., Bao Q. (2013). Identification of differentially expressed proteins of *Arthrospira* (*Spirulina*) *plantensis*-YZ under salt-stress conditions by proteomics and qRT-PCR analysis. Proteome Sci..

[34] Herrera A., Boussiba S., Napoleone V., Hohlberg A. (1989). Recovery of c-phycocyanin from the cyanobacterium *Spirulina maxima*. J. Appl. Phycol..

[35] Bennett A., Bogobad L. (1973). Complementary chromatic adaptation in a filamentous blue-green alga. J. Cell Biol..

[36] Jeffrey S.W., Humphrey G.F. (1973). New spectrophotometric equations for determining chlorophylls a, b, c1 and c2 in higher plants, algae and natural phytoplankton. Biochem. Physiol. Pflanz..

[37] Parsons T.R., Strickland J.D.H. (1963). Discussion of spectrophotometric determination of marine-plant pigments, with revised equations for ascertaining chlorophylls and carotenoids. J. Mar. Res..

[38] Rajauria G., Jaiswal A.K., Abu-Gannam N., Gupta S. (2013). Antimicrobial, antioxidant and free radical-scavenging capacity of brown seaweed *Himanthalia elongata* from western coast of Ireland. J. Food Biochem..

[39] Ganesan A. (2008). The impact of natural products upon modern drug discovery. Curr. Opin. Chem. Biol..

